# Post-migration Social Determinants of Health in Asylum Seekers: A Retrospective Qualitative Study

**DOI:** 10.1007/s10903-025-01763-1

**Published:** 2025-11-07

**Authors:** Tanzilya Oren, Samantha Tham, Celeste Cheung, Andrew Milewski, Gunisha Kaur

**Affiliations:** Weill Cornell Medical College, New York, United States

**Keywords:** Refugee, Social determinants of health, Asylum seeker, Migration

## Abstract

In the United States (U.S.), the number of asylum seekers has increased sixfold in the past decade. Limited research has explored the impact of social determinants of health on asylum seekers. To document evidence of torture and trauma, clinicians in medical-legal asylum clinics conduct Forensic Medical Evaluations (FMEs) according to the standardized United Nations Istanbul Protocol. These evaluations represent an uncommon encounter with the U.S. health system during the multi-year asylum process, during which applicants may not otherwise engage with health systems. This study aimed to determine post-migration factors that influence risk for negative health outcomes in U.S. asylum applicants and to categorize risk factors within the U.S. Department of Health and Human Services’ social determinants of health framework. We performed a qualitative, retrospective study of a representative, purposive sample of forensic medical evaluations from 2010 to 2020 from the Weill Cornell Center for Human Rights’ database. We identified major themes pertaining to post-migration risk and protective factors organized across social determinant domains. The 58 FMEs represented 29 asylum seekers in the U.S. The mean age was 30 years. Of the participants, 55% were female and 45% were male. The sample represented a global population, with origins from the Americas (41%), Africa (45%), and Asia (14%). Our analysis additionally identified the prolonged asylum process as a novel, unique structural barrier and identified protective factors, including community support. Given numerous barriers to accessing care experienced during the asylum process, this study identified a unique opportunity to utilize forensic medical evaluations to screen for social determinants of health.

## Introduction

The forced displacement of people from their homes due to persecution, structural violence, natural disasters, and other causes, has continually increased over the past decade. There are currently more than 123 million forcibly displaced people worldwide as recorded by the United Nations’ most recent estimates in 2024 [1]. Over five million are asylum seekers who reside in a host country while they await an immigration decision on their claims for protection [2]. In the United States (U.S.), the number of asylum filings has increased nearly sixfold between 2010 and 2022 [3].

Multiple studies in the asylum seeking and broader refugee population have identified increased prevalence of adverse physical and mental health outcomes. For example, a 2024 cross-sectional study of 453 asylum seekers in the U.S. found a 94%, 47%, and 50% prevalence of symptoms of psychological stress, cardiovascular disease (CVD), and somatic pain, respectively [4]. Both stress and pain symptoms were independently predictive of CVD symptoms. A 2016 study of 331 Cambodian resettled refugees in the U.S. found this population was more likely to have diabetes, hypertension, and hyperlipidemia when controlled for gender and age [5]. A meta-analysis of mental health outcomes in refugees identified an association between increased torture prevalence and increased prevalence of depression and PTSD [6].

The Social Determinants of Health (SDoH) conceptual framework delineates five key nonmedical factors that influence health outcomes. As defined by the U.S. Department of Health and Human Services (HHS) Healthy People 2030 Initiative and the Centers for Disease Control and Prevention (CDC) [7–12], the main SDoH domains are: (1) Economic stability, (2) Education access and quality, (3) Healthcare access and quality, (4) Neighborhood and built environment, and (5) Social and community context. Others have argued for inclusion of structural determinants—such as systemic discrimination based on identity—as an additional SDoH domain [10–12].

While a vast body of evidence documents the impact of pre-migration experiences (e.g., war, persecution, torture) on post-migration health [6, 13], recent studies have highlighted the significant contribution of post-migration social factors (i.e., while asylum seekers reside in a host country and await immigration decisions) on health outcomes [7, 14–29]. In the U.S., asylum seekers are generally ineligible for legal support and social services, especially at a federal level, including critical determinants of health such as healthcare access or housing support, that resettled refugees are provided. Studies examining post-migration factors thus far have focused on displaced populations resettled in countries outside of the U.S [21, 22, 28, 30]. Literature focused on U.S.-resettled displaced peoples has begun to broadly organize post-migration riskfactors into loose themes, such as “material and economic strain,” “social strain,” “family conflict” [15]. Further investigation is therefore needed to better elucidate post-migration risk factors in the U.S., characterize their relationship within the SDoH framework, and identify the potential intervention opportunity they provide for improving health outcomes.

Under U.S. law, asylum seekers must prove a “well-founded fear of persecution” on account of their race, religion, nationality, political group, or membership in a social group. During a forensic medical evaluation (FME) conducted according to the United Nations Istanbul Protocol (UNIP) to support a legal asylum claim, clinicians record a detailed history of harm and any pertinent previous history; record evidence of physical and psychological sequelae of the harm; and comment on the degree of consistency between clinical findings and the applicants’ narrative of abuse [31, 32]. They may also address the specialized medical or mental health treatment needed and adverse effects if the applicant returned to their home country. Although screening for SDoH is also not a standardized part of the FME, clinicians conducting these evaluations – potentially an asylum seeker’s first and only encounter with U.S. medical professionals – serve a vital role in not only diagnosing physical and psychological symptoms of trauma but also screening for post-migration risk factors [33, 34]. In this study, we aimed to identify post-migration factors in the FMEs that were a risk for, or protective of, negative health outcomes, with subsequent mapping of risk factors onto the HHS SDoH framework.

## Methods

### Study Design and Participants

With approval by Weill Cornell’s Institutional Review Board (IRB), this is a retrospective study of 465 de-identified FMEs from the Weill Cornell Center for Human Rights (WCCHR) Database Registry from 2010 to 2020. Asylum applicants may receive a psychological and/or medical/gynecological evaluation from WCCHR. To capture the most rich and comprehensive review of mental and physical health determinants, 58 FMEs performed for 29 applicants who received both a psychological plus either a medical or gynecological evaluation were purposively selected for coding.

### Data Analysis

Informed by the Consolidated Criteria for Reporting Qualitative Research (COREQ) guideline, qualitative deductive thematic analysis of the retrospective FMEs was conducted using the Dedoose software (version 8.1) [35, 36]. We utilized the HHS Healthy People 2030 framework of SDoH to create the code nodes and identify SDoH-related risk factors in the sections of FMEs detailing post-migration narrative [37].

Two reviewers (T.O. and S.T.) were selected as coders and trained on creating code trees utilizing the Dedoose software. After an initial review of all transcripts, the two coders independently coded each FME to generate major and child codes as related to post-migration factors documenting risk for or protection from negative health outcomes and developed preliminary themes. Through an iterative process, the reviewers compared, re-arranged, and renamed codes; added new codes and themes; and combined all initial codes into broader thematic categories. [37, 38, 39]. We then selected, re-arranged, and renamed codes, subsuming them into unique themes and subthemes. Three authors (T.O, S.T., G.K.) agreed on the code tree with codes pertinent to the research questions and selected representative quotes.

## Results

### Participant Demographic Characteristics

We analyzed 58 FMEs of 29 asylum applicants. The applicants were 55% (*n* = 16) female and 45% (*n* = 13) male. At the time of the evaluation, 21% (*n* = 6) were 16–19 years of age, 58% (*n* = 17) were 20–35 years of age, and 21% (*n* = 6) were 36–59 years of age. The sample represented a global population, with 35% (*n* = 10) from Central/South America, 45% (*n* = 13) from Africa, 13% (*n* = 4) from Asia, and 7% (*n* = 2) from other regions. The time between applicants’ arrival in the U.S. and their latest medical evaluation varied between less than 1 year to 9 years, with an average of 3.2 years. Additional demographic details are noted in Table 1.

**Table 1:** Applicants’ demographic characteristics

Characteristic	Applicants, No. (%) (*N* = 29)
Sex	
Male	13 (44.8)
Female	16 (55.2)
Region of Origin	
North America	1 (3.4)
Central/South America	10 (34.5)
Caribbean	1 (3.4)
East Asia	1 (3.4)
South Asia	3 (10.3)
Northern Africa	1 (3.4)
West Africa	10 (34.5)
Central Africa	2 (6.9)
Age, in No. of years	
16–19	6 (20.7)
20–35	17 (58.6)
36–59	6 (20.7)
Highest level of education	
Less than high school	11 (37.9)
High school	7 (24.1)
Vocational/technical school	3 (10.3)
Bachelors	5 (17.2)
Unknown	3 (10.3)
Interview Language	
English	11 (37.9)
Limited English	4 (13.8)
Spanish	8 (27.6)
French	4 (13.8)
Punjabi	1 (3.4)
Chinese	1 (3.4)
Household Size in the U.S., No.	
0	2 (6.9)
1	5 (17.2)
2	7 (24.1)
3	6 (20.7)
4+	9 (31.0)
Time Between U.S. Arrival and Recent Interview, in years	
0–1	9 (31.0)
2–5	14 (48.3)
6–9	5 (17.2)
Unknown	1 (3.4)
Occupation Before U.S. Arrival	
Student	8 (27.6)
Non-profit/Advocacy	3 (10.3)
Employee at Local Store	3 (10.3)
Construction/Engineering	2 (6.9)
Government employee	1 (3.4)
Business Owner	1 (3.4)
Accounting Assistant	1 (3.4)
Delivery	1 (3.4)
Fare Collector	1 (3.4)
Farmer	1 (3.4)
Pastor	1 (3.4)
Driver	1 (3.4)
Unknown	5 (17.2)

### Post-Migration Risk Factors Within SDoH Framewor

**Fig. 1:**
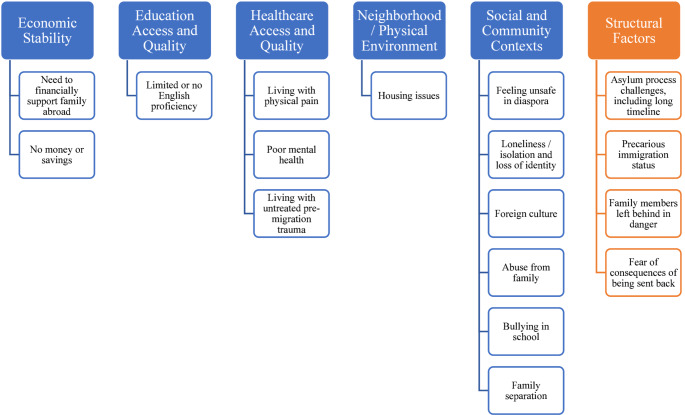
Post-migration risk factors identified and mapped onto the HHS-defined SDoH framework, with addition of structural factors

Within the HHS-defined SDoH framework, 13 codes labeling post-migration risk factors experienced by the asylum applicants could be categorized across the five predefined main domains (Fig. 1). The most frequently mentioned predefined domain was healthcare access and quality, with 21 applicants noted to have poor mental health. Education access was the next most frequently mentioned SDoH risk factor: 18 applicants were determined to have limited to no English proficiency based on clinician assessment and utilization of interpreter. The third most frequently mentioned SDoH risk factor was feelings of isolation and loneliness, with nine applicants describing its negative health impact. The prolonged asylum process impacted the health of this population, as documented by the presence of at least one of the five codes across all 29 applicants. Representative quotes from the FMEs are provided in Table 2.

**Table 2:** Post-migration risk factors theme and subthemes with representative clinician quotes

Theme	Subtheme	Representative Clinician Quote
Economic Stability	Limited ability to financially self-support	Impoverished, he will occasionally get some work washing dishes or cleaning at a local restaurant so that he can buy food and pay for his room; but he is generally dependent on the generosity of his housemates and acquaintances.
	Not having work authorization	He does not have work authorization and does not work; however, he reports that he is active with the foundation in [redacted city], which helped him obtain his visa.
	Working in a survival low-paying job	When she arrived in the United States, she was able to find a position at a hair-braiding salon that taught her to braid hair. When the salon closed, she started walking outside looking for new work and was able to find a position filling in at another hair-braiding salon when the salon needs extra help. She has also subsequently received a social security number and a work permit and has been applying for many jobs, largely unsuccessfully. She has found some temporary positions as a live-in aide with elderly clients in [redacted state] through an agency and has worked approximately five such jobs. She is very motivated to work to pay for rent, her phone bill, and food.
Education Access and Quality	Low English proficiency	Alone in a foreign culture, without English language skills, and with no money, Mr. X has received no treatment for his illnesses and would likely benefit from psychotherapy and antidepressant medication to help resolve disabling symptoms, promote a better adaptation to the dramatic upheavals in his life, and enhance recovery from his traumas.
Healthcare Access and Quality	Lack of access to healthcare	He has never received any medical care in the United States. He visited an emergency room in [redacted city] following a motor vehicle accident, with a likely concussion. He was denied care due to his lack of health insurance.
	Unfamiliarity with mental health care	Despite having continuing symptoms, she has never sought any help,largely due to her unfamiliarity with health care, psychological treatment or conditions, and a reluctance to reveal herself to others.
	Stigma around mental health care	He did not accept offered (free) psychiatric treatment, saying that he didn’t think it would help, that he wouldn’t want to jeopardize his asylum case by using resources that someone else might need, and he does not want to think of himself as “mental.” He prefers his use of alcohol (he described drinking 4 beers daily and using it to get sleep) as this is more socially acceptable than seeing a psychiatrist.
	Living with chronic pain	A detailed review of the systems revealed that she continues to have pain since her surgery. In the initial period after the injury, pain in both arms was constant and unrelenting. Now it is still present but varies in intensity. She has pain in her left wrist and right arm at the location of injury with changes in temperature, exercise, or movement. The right arm pain is described as internal, radiating, and shooting up her arm from her fingertips to the shoulder. She denies any numbness or paresthesia. At times this pain is 1–3/10 and at other times 8–9/10 and unbearable. She only uses over-the-counter pain medicine to treat this pain.
	Living with poor mental health	He has experienced depressed mood; disturbed sleep and loss of appetite leading to significant weight loss; intrusive thoughts and nightmares about his circumstances; and heightened anxiety and nervousness. He is often anxious, worried, and prone to obsessively ruminating about his life. Mr. X has received no treatment for his condition and would likely benefit from treatment. He states that these symptoms were and still are triggered by the smell of smoke and urine and sounds of footsteps, which remind him of his past trauma. Mr. X states that he has felt an acute exacerbation of those symptoms during his immigration proceedings as he repeatedly recounts these traumas.
Neighborhood/Physical Environment	Living in unstable housing	He works from 5 PM − 12 AM, 35 h a week, in a restaurant near [redacted neighborhood]. He would like to find a different living arrangement and is looking for a separate apartment as he does not feel comfortable sharing a room with 2 other people.
Social and Community Contexts	Isolation and loneliness	Since moving to the United States, she reports feeling depressed at least 80% of the time. She is ashamed and humiliated by her past to the point where she believes she disturbs people around her when she accidentally touches them (as on a public bus) or is overheard speaking in her native language. She experiences intrusive thoughts, frequent nightmares, painful thoughts about death and dying, and profoundly low self-esteem. Many of her symptoms were present while she was still in [redacted country], but she had a full-time job and school (which she loved) and was distracted from her underlying torment. Since she has arrived here, she has not had distractions from her thoughts and her isolation and loneliness have compounded her inner struggles.
Structural Factors	Separation from family	She still lives in fear that [redacted group] might harm her and her family here and mourns for her two daughters who she was forced to leave behind in [redacted country]. Mr. X reports feeling sometimes anxious and fearful, but mostly sad. He has no contact with his family in [redacted country].
	The long and onerous asylum process	He states that this job is isolating and that he constantly thinks about how his delayed immigration status has “ruined his life” and how he “has lost his dignity.” Mr. X also states that customers are often stressful, and he recalled one passenger in particular who refused to pay his fare and then tried to assault Mr. X. Mr. X knew to restrain himself as any such skirmish could jeopardize his asylum case, even though he was in the right. Incidents like this make him feel that he “has no dignity left in this country.” He lost 10 pounds in the past month after learning of his case’s postponement. He has lost weight at other times too as he often is so upset that he “forgets to eat”.
	Asylum denials	He had heard that this translator was very good, but during the interview, he realized that this was not the case and that the man was giving very brief and terse answers as translations of complicated things that Mr. X was trying to convey. Even though he was told the interview went well, he later received a “removal” letter. She reports that much of her anxiety stems from the fear of being sent back to [redacted country] after all they have been through. She states that she can’t imagine going through the deportation process again and is anxious about being able to provide for and protect her children.
	Fear of being sent back	Mr. X is afraid of the possibility of not being allowed to remain in the United States. If he were forced to return to [redacted country] he fears that he will be subjected anew to the life-long harassment, social rejection, and violence targeted at his homosexual orientation that he has experienced since a young age in his country. He fears that the harassment will be worse than before because of his recent HIV-positive status. Ms. X denies currently being afraid for her life in the U.S., but she experiences mortal fear at the thought that she may have to go back to [redacted country]. She is afraid of returning to [redacted country] and says that she “prefers to die” rather than go back there.
	Family facing danger abroad	Their oldest daughter stayed in [redacted country], but he does not know where she is or even if she is alive. The most troubling thing for him was that his wife and children are at home and alone in [redacted country].

#### Domain 1: Economic Stability

Owing to their status as asylum applicants, all individuals had limited or no access to financial aid, including Supplemental Food Assistance Program (SNAP, i.e., food stamps), public assistance, and other social welfare benefits. Most worked in positions in cleaning, construction, retail, and driving. At least seven applicants reported working physically demanding jobs with unpredictable hours that did not make use of their professional qualifications. One individual had been attending college in his home country before fleeing to the United States and was currently working “from 5PM – 12AM, 35 hours a week, in a restaurant near [redacted neighborhood].” Another applicant reported an inability to work because “he feels ‘sick inside’ when he stands for more than one hour. He went on to describe this sickness as right flank pain and headaches.”

#### Domain 2: Education Access and Quality

As determined by the clinician’s informal assessment and/or the documented use of an interpreter in the FME, ten applicants had adequate English proficiency, four had limited English proficiency, and 14 did not speak English. Generally, the education attainment among the applicants varied, from not completing high school (*n* = 11) to having a college degree from their home country (*n* = 5). Most applicants described their plans to continue to learn English, attend college, and obtain employment.

#### Domain 3: Healthcare Access and Quality

Asylum seekers who had acute health issues were able to get specialized care, such as scar revision surgery on the face and skull, emergency care for asthma attacks, and HIV treatment. In addition, three of seven applicants who accessed health care received mental health interventions for Post-Traumatic Stress Disorder (PTSD) and suicidal ideation. However, aside from emergency assistance, seven asylum seekers did not seek follow-up medical care despite chronic medical or psychiatric conditions, highlighting a lack of continuity of care. One FME noted an applicant “has not had any medical care since arriving in United States, but did relate several current symptoms.” Ten applicants did not access any healthcare. At least two did not have health insurance; four were not familiar with mental health care or were reluctant to seek this care, with one individual noting an explicit stigma around mental health as“he does not want to think of himself as ‘mental’”. Eight applicants lived with ongoing and largely untreated chronic pain, and almost all applicants (*n* = 25) lived with untreated poor mental health, with clinicians recommending further psychiatric care.

#### Domain 4: Neighborhood/Physical Environment

Sixteen applicants mentioned unstable housing situations: three individuals lived in homeless shelters, seven lived with relatives other than the immediate family, three shared rooms with strangers, and three rented rooms with roommates. Despite the potential increased stability in living with immediate family members, one individual described a physically and verbally abusive relationship because “her mother is not happy that she’s there…instead her mother calls her vulgar names and has hit her once.”

#### Domain 5: Social and Community Contexts

Although more than half of the applicants reported feeling safe in the U.S., three individuals mentioned that they did not feel safe in the diaspora of immigrants from their own countries. Nine applicants also mentioned loneliness, isolation, and loss of identity post-migration, experiences that arose from mental or physical health issues and from a need to survive independently. One such individual said that “he does not feel that he has adequate social support or a community to which he belongs”. Many applicants (*n* = 10) endured separation from family and could not travel due to immigration proceedings, resulting in increased feelings of loneliness and anxious preoccupations.

#### Proposed Domain 6: Structural Factors Related To Lengthy Asylum Process

The asylum process was found to be a structural determinant of health that affected all 29 applicants, with the immigration application backlog resulting in a prolonged, uncertain, or unstable immigration status. Most applicants (*n* = 20) relayed moderate to severe levels of increased anxiety at potential deportation. Twelve applicants explicitly described their fear of being deported given the potential for abuse, repeated torture, and death upon returning home. One applicant stated that he “fears that he will be subjected anew to the life-long harassment, social rejection, and violence targeted at his homosexual orientation that he has experienced since a young age in his country.” Five individuals were denied asylum in another country or during a primary application for asylum in the U.S. One such individual reported that “much of their anxiety stems from the fear of being sent back to [redacted] after all they have been through” as she and her two children had already undergone the traumatic experience of deportation from a different host country. Four applicants detailed their frustration and limitations of the liminal and precarious immigration status of an asylum seeker, including being in a persistent negative emotional state of frustration and not accepting medical treatment due to perceived fear of punitive immigration policies. One applicant described the detrimental effects of repeatedly recounting his experiences during proceedings as he felt an acute exacerbation of nightmares and hopelessness. Another applicant reported losing ten pounds in the month after he learned of his case’s postponement. An additional consequence of the complex and protracted asylum proceedings was prolonged separation from family, as spouses and children of ten applicants were still residing in their home countries. These ten applicants expressed sadness, loneliness, and worry over the safety of their family members.

### Post-migration Protective Factors

A vital aspect of asylum applicants’ post-migration experiences was protective factors, including development of coping strategies, to mitigate the detrimental effects of risk factors. Representative quotes of protective factors are listed in Table 3. The clinicians noted mentions of future plans and aspirations for a better life for 14 applicants. Having a support system through relatives, friends, churches, and organizations in the U.S. was another protective factor. Spirituality, going to church or mosque, and praying were coping strategies for at least six applicants. Religion served as a particularly strong mitigator of risk as one applicant noted that he “denied any thoughts of suicide as it is against his religious beliefs.” Although many applicants were separated from their immediate families, having a partner in the U.S. (*n* = 4) improved their mental state. Engaging in sports and hobbies (*n* = 3), learning English in classes (*n* = 3), and meeting people speaking the same language or from the home region (*n* = 2), helped others. At least five applicants also noted walking, reading, using traditional healing methods, drawing, and providing testimony, as protective factors.

**Table 3:** Post-migration protective factors themes with representative clinician quotes

Theme	Representative Clinician Quote
Having plans, goals and dreams of the future	Also, Mr. X is very focused on achieving personal goals (getting an education and “using what’s in my head” to make a living), taking care of his mother, and giving back to the country where he feels safe. These goals can help people be focused and more grounded in their present life, and therefore can be somewhat protective against developing PTSD.
Support system	He is well integrated in the community; he attends church twice per week, Sundays and Monday evening services at [redacted] Church. He plays soccer and swims once per week. And is looking forward to attending summer camp from his Church. He also expressed interest in working at a restaurant during the summer.
Praying/Spirituality	He has these memories primarily when he is alone and is “thinking too much.” He therefore prefers being around people. Praying at night helps him go to sleep in spite of the bad memories, and he normally goes to sleep in the evenings after praying.
Having a partner in U.S.	She is living with the man who fathered her son, and it seems to be a loving relationship that may restore her trust. She is a good mother, getting satisfaction from caregiving and able to make considerable sacrifices to improve her children’s lives allowing her daughters to live with her husband so they could have a better education, and moving to the U.S. to save the life of her son.
Hobbies/interests	She is able to take pleasure in caring for others and in cooking. And she has taken up running, which she blurted out with pride.
Learning English	He has made great progress learning the English language
Meeting people from own culture	She reported having no close friends in the U.S. but stated that she has recently become acquainted with other French-speaking African women in [redacted city].
Other ways of coping	He attempts to read, to learn some English, and often takes long walks to try to clear his head. Although clearly brutally traumatized, Mr. X seems to have been able to resume his professional and personal life. He received promotions and recognitions for his work as a civil engineer, being given increased supervisory responsibilities.

## Discussion

This first in-kind retrospective, qualitative, thematic analysis of 58 psychological and physical (medical/gynecological) FMEs identified post-migration, HHS-defined SDoH risk factors experienced by asylum applicants in the U.S. We found post-migration risk factors that corresponded with all five HHS-defined domains. We also identified and propose a new structural factors domain, which includes subthemes of a prolonged complex asylum process, unique to this population (Fig. 1). Inclusion of this novel domain is appropriate given robust, emerging research on the negative impact of punitive immigration policies on physical and mental health [40, 41]. Our analysis found that each of these factors, within the HHS-defined domains andexternal to them, negatively impacted physical and mental health of U.S. asylum applicants [21–23, 28, 29, 42], but its analysis of risk factors within a widely accepted, standardized SDoH framework demonstrates the potential clinical and research utility of FMEs.

The increased acknowledgement of SDoH as a strong predictor of health outcomes has led U.S. health systems to implement internal programs, external partnerships, and interventions to improve health [43–50]. Currently, many active screening tools have been developed to identify social needs in vulnerable populations, such as Medicaid/Medicare dual-eligible beneficiaries. Within displaced populations, limited tools, such as the refugee post-migration stress scale (RPMS), have been developed to assess the level of post-migration stress [24]. Although the domains used in the RPMS overlap with HSS-defined SDoH, the tool is not intended to screen for social needs.

The significant contribution of SDoH risk factors to poor physical and mental health outcomes in U.S. asylum seekers, identified here, represents an opportunity for intervention. The UNIP-based FME is primarily a tool to identify and document how a person’s narrative of trauma is corroborated by physical and psychological evidence in their asylum application [31–34]. However, given that it is uncommon for asylum seekers to interact with the healthcare system, FMEs provide a unique opportunity to identify SDoH risk factors that influence a person’s health and wellbeing while they await an asylum decision. Evidence of improved health outcomes after using social screening tools in other high-risk populations and the significant impact of SDOH on health outcomes strongly argue for the development of a tailored tool [49]. One potential tool could include adapting the 5 main and 8 supplemental domains in the Accountable Health Communities Health-Related Social Needs Screening Tool for FMEs, as they already capture many of the post-migration risk factors identified in this study [51]. Another option is to develop a customized tool for this unique, vulnerable, and rapidly growing patient population.

Limited evidence in trauma-exposed populations suggest that certain adaptive coping strategies may play a buffering role for trauma exposure and can be beneficial for their wellbeing [52]. Specifically, problem-solving and cognitive restructuring strategies have been identified as a moderator in experiencing less psychological distress in a meta-analysis of trauma-exposed populations [52]. However, the meta-analysis did not find a significant association between an emotional coping approach, including social support, and less distress. Our study identified several protective factors, such as forming aspirations or integrating into established community networks, that fall within different types of coping strategies, including an emotional coping approach. Further studies investigating the broad spectrum of coping strategies, such as the mediating effects of emotional or social-seeking strategies on post-migration stress reduction, present an important area of further research.

### Limitations

There are several limitations of this study, including the retrospective design, indirect clinicians-only reports, and nonrepresentative sample of the entire population of U.S. asylum applicants. However, this first-in-kind analysis of the written UNIP-based FMEs provides valuable insights, including themes to be further studied in prospective and longitudinal studies. This study can also inform future in-depth, qualitative, and interview-based studies as well as larger quantitative studies that examine the effects of post-migration SDoH or test novel interventions.

### New Contribution To the Literature

In this study, we identified social, economic, and structural barriers to accessing healthcare for asylum seekers. Notably, we identified the complex and prolonged application process as a novel, modifiable factor impacting the health of U.S. asylum applicants. While the CDC recommends that SDoH be considered in the health screening of resettled refugees domestically [53], a standardized, validated SDoH screening for asylum seekers is yet to be developed and deployed. Given the limited healthcare engagement of this population during the years that they await their immigration application decision, our findings demonstrate the research potential of using FMEs to investigate questions related to asylum seeker health and the opportunity to integrate SDoH screening for U.S. asylum applicants in the FME process.

## Data Availability

No datasets were generated or analysed during the current study.
